# Structures and cytotoxicities of three new sesquiterpenes from cultures of *Armillaria* sp.

**DOI:** 10.1007/s13659-012-0077-1

**Published:** 2012-12-20

**Authors:** Xia Yin, Tao Feng, Ji-Kai Liu

**Affiliations:** 1 State Key Laboratory of Phytochemistry and Plant Resources in West China, Kunming Institute of Botany, Chinese Academy of Sciences, Kunming, 650201 China; 2 University of Chinese Academy of Sciences, Beijing, 100049 China

**Keywords:** basidiomycete, *Armillaria* sp., sesquiterpene aryl ester, cytotoxicities

## Abstract

Three new sesquiterpene aryl esters, named 10-dehydroxy-melleoliede B (**1**), 1-*O*-formyl-10-dehydroxy-melleoliede B (**2**) and 10-oxo-melleoliede B (**3**) together with six known ones (**4**–**9**), were isolated from the cultures of *Armillaria* sp. The structures of the new compounds were elucidated based on the extensive spectroscopic methods. Compounds **1**, **2**, and **5**–**9** exhibited moderate cytotoxicities.

**Electronic Supplementary Material:**

Supplementary material is available for this article at 10.1007/s13659-012-0077-1 and is accessible for authorized users.

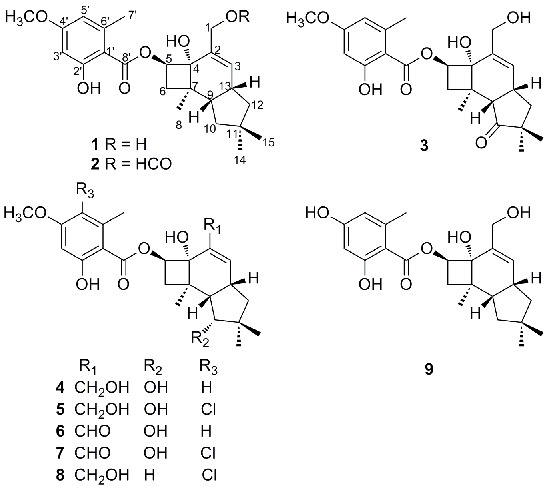

## Introduction

The basidiomycete genus *Armillaria* is recognized for the production of rich secondary metabolites with structure diversities as well as interesting biological activities.[1] Among those metabolites, sesquiterpene aryl esters are quite a big family which showed antibacterial, antifungal activities,[1c,1j] and cytotoxicity.[1o,1p] As a part of our search for naturally occurring secondary metabolites with diverse structures from fungi in China,[2] we have carried out the chemical investigation of *Armillaria* sp. cultures, which led to the isolation of three new sesquiterpene aryl esters, named 10-dehydroxy-melleoliede B (**1**), 1-*O*-formyl-10-dehydroxy-melleoliede B (**2**), and 10-oxo-melleoliede B (**3**), together with melleolide B (**4**),[1g] 5'-chloromelleolide (**5**),[1g] armillarigin (**6**),[1g] armillarikin (**7**),[1g] A52a (**8**),[3] and armillane (**9**).[1p] Those new compounds were elucidated by means of spectroscopic methods, while the known compounds were identified by comparison with data in the literature. All of these compounds were evaluated for cytotoxicity against five human cancer cell lines.

## Results and Discussion

Compound **1**, obtained as a colorless oil, had the molecular formula of $C_{24}H_{32}O_6$ based on the negative-ion HRESIMS at *m/z* 415.2118 $[M - H]^-$ (calcd for $C_{24}H_{31}O_6$, 415.2120). The IR data exhibited absorption bands for a hydroxy at 3430 $cm^{-1}$, an ester group at 1646 $cm^{-1}$ and a substituted aromatic ring at 1617 $cm^{-1}$ and 1578 $cm^{-1}$,[1] which consisted with the UV spectra. The $^1H$ NMR spectrum (Table 1) of **1** exhibited one chelated phenolic hydroxy proton at $\delta_H$ 11.63, two aromatic protons at $\delta_H$ 6.31, one methoxy group at $\delta_H$ 3.80, four methyls including an aromatic one at $\delta_H$ 2.41. The $^{13}C$ NMR spectrum revealed 24 carbons resonances (Table 2), which were classified into five methyl groups (three aliphatic, one oxygenated and one aromatic), four aliphatic methylenes (one oxygenated), six methines (three olefinic), and nine quaternary carbons (one carbonyl), indicating the presence of a tetra-substituted benzene unit as well as one ester carbonyl signal at $\delta_C$ 171.8. The above data were quite closely related to those of melleolide B (**4**).[1g] The significant differences of the $^{13}C$ NMR data between **1** and **4** were the lack of a secondary alcohol

*To whom correspondence should be addressed. E-mail: jkliu@mail.kib.ac.cn

**Table 1. $^1$H NMR data of 1–3 (1, 3 in $Me_2CO$-$d_6$ ; 2 in $CDCl_3$)**

| | 1 | 2 | 3 |
|---|---|---|---|
| No. | $\delta_H$ (*J* in Hz) | $\delta_H$ (*J* in Hz) | $\delta_H$ (*J* in Hz) |
| 1 | 4.31, br. d (12.3); 4.03, dd (12.3, 2.9) | 4.72, d (12.3); 4.60, d (12.3) | 4.29, br. d (13.6); 4.00, br. d (13.6) |
| 3 | 5.77, br. s | 5.90, br. s | 5.98, br. s |
| 5 | 5.64, t (8.7) | 5.48, t (8.7) | 5.67, t (8.6) |
| 6 | 1.94, dd (11.2, 8.7); 1.70, dd (11.2, 8.7) | 1.97, dd (11.1, 8.7); 1.77, dd (11.1, 8.7) | 1.95, dd (11.3, 8.7); 1.73, dd (11.3, 8.7) |
| 8 | 1.25, s | 1.28, s | 1.64, s |
| 9 | 2.19, m | 2.19, m | 2.64, d (7.5) |
| 10 | 1.45, overlapped; 1.38, overlapped | 1.44, overlapped; 1.24, overlapped | |
| 12 | 1.86, dd (13.2, 8.5); 1.50, overlapped | 1.85, dd (13.4, 8.7); 1.48, overlapped | 2.09, overlapped; 1.91, br. d (13.0) |
| 13 | 2.78, br. t (7.8) | 2.78, br. t (7.7) | 3.13, br. t (7.5) |
| 14 | 1.00, s | 0.95, s | 0.99, s |
| 15 | 1.00, s | 0.99, s | 0.97, s |
| 3' | 6.31, br. s | 6.32, d (2.4) | 6.31, br. s |
| 5' | 6.31, br. s | 6.26, d (2.4) | 6.32, br. s |
| 7' | 2.41, s | 2.40, s | 2.43, s |
| $OCH_3$ | 3.80, s | 3.79, s | 3.81, s |
| Ar-OH | 11.63, s | 11.61, s | 11.60, br. s |
| CHO | | 7.87, s | |

signal and the presence of a methylene signal at $\delta_C$ 41.9 in **1**, revealing the absence of HO-10 in **1**. The above assumption was further confirmed by the $^1$H-$^1$H COSY correlations of H-10/H-9/H-13/H-12, and the HMBC correlations from H-10 to C-9, C-11, and C-13 (Figure 1).

The ROESY correlations (Figure 1) of H-9/H-13, H-9/H-6*β*, H-5/H-6*α*, H-5/H-8, and H-6*α*/H-8 of **1**, as well as the similar chemical shifts of the chiral carbons comparing with those of melleolide B, suggested the *cis-anti-cis* stereochemistry of the three rings.[4] Therefore, the structure of compound **1** was established as 10-dehydroxy-melleoliede B.

**Figure 1.** Key HMBC, $^1$H–$^1$H COSY, and ROESY correlations for **1**

Compound **2**, purified as a colorless oil, had a molecular formula of $C_{25}H_{32}O_7$ according to its negative-ion HRESIMS at *m/z* 443.2070 ($[M - H]^-$, calcd, 443.2069). The IR spectrum showed the presence of a hydroxy group at 3432 $cm^{-1}$ and an ester moiety at 1629 $cm^{-1}$.The 1D NMR (Tables 1 and 2) data were quite similar with those of **1** except for an additional signal for a formyl group ($\delta_H$ 7.87, $\delta_C$ 160.8). The formyl group attached at C-1 was confirmed by the HMBC of H-1 to C-CHO. Detailed analysis of 1D and 2D NMR data suggested that the other parts of **2** were the same to those of **1**. Therefore, compound **2** was established as 1-*O*-formyl-10-dehydroxy-melleoliede B.

Compound **3**, was obtained as a colorless oil. The negative-ion HRESIMS afforded the molecular formula of $C_{24}H_{30}O_7$ at *m/z* 429.1917 ($[M - H]^-$, calcd, 429.1913). The IR data exhibited absorption bands for a hydroxy at 3431 $cm^{-1}$, an ester group at 1642 $cm^{-1}$ and a substituted aromatic ring at 1618 $cm^{-1}$ and 1579 $cm^{-1}$, which were similar with those of compound **1**. Detailed analysis of the $^{13}$C NMR suggested that the main difference between **3** and **1** was that a methine was oxidized into a keto carbonyl group at $\delta_C$ 220.6 (C-10), which resulted in the downfield shift of C-9 ($\Delta\delta$ 7.1 ppm), C-11 ($\Delta\delta$ 6.2 ppm) in compound **3**. The above conclusion was supported by the HMBC correlations of H-9, H-13, and H-14 to C-10, and H-3 ($\delta_H$ 5.98) to C-12 ($\delta_C$ 41.9). Further analysis of 1D and 2D NMR data and coupling constants suggested that the other parts of the molecular was the same to compound **1**. Thus, compound **3** was established as 10-oxo-melleoliede B.

All compounds were evaluated for their cytotoxicity against five human cancer cell lines using the MTT method as reported previously.[5] Compounds **1**, **2**, and **5–9** showed cytotoxicities as shown in Table 3. It is notable that compound **2** showed stronger cytotoxicities than that of the positive control in MCF-7 and SW480 cell lines.

**Table 3. Cytotoxicity for compounds 1–9 ($IC_{50}$, $\mu$M)**

| Entry | HL-60 | SMMC-7721 | A-549 | MCF-7 | SW480 |
|---|---|---|---|---|---|
| **1** | 17.79 | 20.90 | 16.79 | 16.49 | 17.44 |
| **2** | 14.50 | 23.16 | 18.41 | 5.34 | 10.77 |
| **3** | > 40 | > 40 | > 40 | > 40 | > 40 |
| **4** | > 40 | > 40 | > 40 | > 40 | > 40 |
| **5** | > 40 | > 40 | 20.11 | 30.06 | > 40 |
| **6** | 14.27 | 19.51 | 10.01 | 10.67 | 19.19 |
| **7** | 14.11 | 17.41 | 14.73 | 11.52 | 19.89 |
| **8** | 17.06 | 17.77 | 15.89 | 14.10 | 15.70 |
| **9** | 20.27 | 30.36 | 16.62 | 16.56 | > 40 |
| cisplatin[a] | 1.3 | 15.4 | 17.3 | 15.8 | 24.4 |

[a]Cisplatin was used as positive control.

**Table 2.** $^{13}C$ **NMR data of 1–3 (1, 3 in $Me_2CO$-$d_6$ ; 2 in $CDCl_3$)**

| | 1 | 2 | 3 |
|---|---|---|---|
| No. | $\delta_C$ mult | $\delta_C$ mult | $\delta_C$ mult |
| 1 | 65.2, t | 64.1, t | 63.9, t |
| 2 | 135.2, s | 130.2, s | 140.3, s |
| 3 | 132.8, d | 137.9, d | 131.0, d |
| 4 | 77.6, s | 77.6, s | 77.7, s |
| 5 | 79.4, d | 77.9, d | 79.4, d |
| 6 | 33.4, t | 32.2, t | 34.7, t |
| 7 | 39.4, s | 38.5, s | 39.3, s |
| 8 | 22.0, q | 21.7, q | 20.4, q |
| 9 | 45.3, d | 44.0, d | 52.4, d |
| 10 | 41.9, t | 41.5, t | 220.6, s |
| 11 | 38.4, s | 37.9, s | 44.6, s |
| 12 | 48.4, t | 47.4, t | 41.9, t |
| 13 | 39.9, d | 39.2, d | 35.0, d |
| 14 | 32.3, q | 31.8, q | 28.2, q |
| 15 | 32.2, q | 31.7, q | 27.4, q |
| 1' | 105.9, s | 104.6, s | 105.9, s |
| 2' | 166.3, s | 165.7, s | 166.4, s |
| 3' | 99.5, d | 98.8, d | 99.5, d |
| 4' | 165.0, s | 164.2. s | 165.5, s |
| 5' | 111.6, d | 111.3, d | 111.5, d |
| 6' | 144.1, s | 143.0, s | 144.3, s |
| 7' | 24.2, q | 24.2, q | 24.3, q |
| 8' | 171.8, s | 171.3, s | 171.8, s |
| $OCH_3$ | 55.7, q | 55.3, q | 55.8, q |
| CHO | | 160.8, d | |

## Experimental Section

**General Experimental Procedures.** Optical rotations (OR) were recorded on a Jasco P-1020 digital polarimeter. UV and the IR spectra were obtained on a Shimadzu UV2401PC and a Bruker Tensor 27 FT-IR spectrometer with KBr pellets. Nuclear Magnetic Resonance (NMR) spectra were obtained on a BRUKER AM-400 and a BRUKER DRX-500 MHz spectrometer with tetramethylsilane (TMS) as an internal standard at room temperature. High-resolution (HR) ESIMS were recorded on an API QSTAR Pulsar spectrometer. Silica gel (200–300 mesh, Qingdao Marine Chemical Ltd., China) and Sephadex LH-20 (Amersham Biosciences, Sweden) were used for open column chromatography (CC). Preparative HPLC was performed on an Agilent 1100 liquid chromatography system equipped with a Zorbax SB-C18 column (9.4 mm × 150 mm). Fractions were monitored by TLC. Spots were visualized by heating silica gel plates immersed in Vanillin-$H_2SO_4$ in ethanol.

**Fungal Material and Cultivation Conditions.** The fungus *Arimillaria* sp. was collected from Wuding, in Yunnan Province, China, in 2005. The culture medium consisted of glucose (5%), peptone from porcine meat (0.15%), yeast powder (0.5%), $KH_2PO_4$ (0.5%) and $MgSO_4$. Fermentation was carried out on a shaker at 160RPM for 25 days.

**Extraction and Isolation.** The culture broth (21 L) was filtered, and the filtrate was extracted three times with ethyl acetate, while the mycelium was extracted three times with $CHCl_3$-MeOH (1:1). The EtOAc layer together with the mycelium extraction was concentrated under reduced pressure to give a crude extract (20 g), and this residue was chromatographed on silica gel (200–300 mesh) column eluted with a gradient of $CHCl_3$-MeOH (1:0 → 0:1) to obtain six fractions (1–6). Fraction 4 was subjected to a silica gel CC (petroleum ether-EtOAc, 6:1) to give 4 sub fractions (4a–4d). Subfraction 4c was further purified by Sephadex LH-20 column (eluted with acetone) to give **2** (6 mg) while 4d was subjected to Sephadex LH-20 column (eluted with $CHCl_3$-MeOH, 1:1) followed by semipreparative HPLC (MeCN-$H_2O$, eluting from 55:45 to 100:0 for 40 min with a flow rate of 10 mL/min) to yield **1** (14 mg) and **3** (11 mg).

**10-Dehydroxy-melleoliede B (1):** colorless oil; $[\alpha]_D^{15}$ + 41.4 (*c* 1.08, MeOH); UV (MeOH) $\lambda_{max}$ (log $\varepsilon$) 302 (3.73), 264 (4.13), 215 (4.35) nm; IR (KBr) $\nu_{max}$ 3430, 2949, 2864, 1646, 1617, 1578, 1444, 1257, 1160 $cm^{-1}$; $^1H$ (400 MHz) and $^{13}C$ NMR (100 MHz) data ($Me_2CO$-$d_6$), see Tables 1 and 2; negative ion HRESIMS *m/z* 415.2118 $[M - H]^-$ (calcd for $C_{24}H_{31}O_6$, 415.2120).

**1-*O*-Formyl-10-dehydroxy-melleoliede B (2):** colorless oil; $[\alpha]_D^{15}$ + 29.4 (*c* 0.29, MeOH); UV (MeOH) $\lambda_{max}$ (log $\varepsilon$) 302 (3.72), 264 (4.12), 215 (4.35) nm; IR (KBr) $\nu_{max}$ 3432, 2920, 1629 $cm^{-1}$; $^1H$ (400 MHz) and $^{13}C$ NMR (125 MHz) data ($CDCl_3$), see Tables 1 and 2; negative ion HRESIMS *m/z* 443.2070 ($[M - H]^-$, calcd 443.2069).

**10-Oxo-melleoliede B (3):** colorless oil; $[\alpha]_D^{15}$ – 42.5 (*c* 0.28, MeOH); UV (MeOH) $\lambda_{max}$ (log $\varepsilon$) 302 (3.70), 265 (4.10), 215 (4.33) nm; IR (KBr) $\nu_{max}$ 3431, 2928, 1730, 1642, 1618, 1579, 1256, 1160 $cm^{-1}$; $^1H$ (400 MHz) and $^{13}C$ NMR (100 MHz) data ($Me_2CO$-$d_6$), see Tables 1 and 2; negative ion HRESIMS *m/z* 429.1917 ($[M - H]^-$, calcd 429.1913).

**Cytotoxicity Assay.** Five human cancer cell lines, breast cancer MCF-7, hepatocellular carcinoma SMMC-7721, human myeloid leukemia HL-60, colon cancer SW480, and lung cancer A-549 cells, were used in the cytotoxic assay. All the cells were cultured in RPMI-1640 or DMEM medium (Hyclone, USA), supplemented with 10% fetal bovine serum (Hyclone, USA) in 5% $CO_2$ at 37 °C. The cytotoxicity assay was performed according to the MTT (3-(4,5-dimethylthiazol-2-yl)-2,5-diphenyl tetrazolium bromide) method in 96-well microplates.[5] Briefly, 100 $\mu$L adherent cells were seeded into each well of 96-well cell culture plates and allowed to adhere for 12 h before drug addition, while suspended cells were seeded just before drug addition with initial density of $1 \times 10^5$ cells/mL. Each tumor cell line was exposed to the test compound dissolved in DMSO at concentrations of 0.0625, 0.32, 1.6, 8, and 40 $\mu$mol in triplicates for 48 h, with cisplatin (Sigma, USA) and vinorelbine (National Institute for the Control of Pharmaceutical and Biological Products, China) as positive controls. After compound treatment, cell viability was detected and a cell growth curve was graphed. $IC_{50}$ values were calculated by Reed and Muench's method.[6]

## Electronic Supplementary Material

Supplementary material is available in the online version of this article at http://dx.doi.org/ 10.1007/s13659-012-0077-1 and is accessible for authorized users.

## Acknowledgments

This project was supported by the National Basic Research Program of China (973 Program, 2009CB522300), the National Natural Sciences Foundation of China (30830113, U1132607).

**Open Access** This article is distributed under the terms of the Creative Commons Attribution License which permits any use, distribution, and reproduction in any medium, provided the original author(s) and source are credited.

## Electronic supplementary material

Supplementary material, approximately 794 KB.
