# Supplementary material for: Structures and cytotoxicities of three new sesquiterpenes from cultures of Armillaria sp
Source: Nat Prod Bioprospect. 2012 Dec 20;2(6):245–8. doi: 10.1007/s13659-012-0077-1 (PMC4131607; doi:10.1007/s13659-012-0077-1)
Supplement: Supplementary file 1 — Supplementary material, approximately 794 KB. [file 13659_2012_77_MOESM1_ESM.pdf]

Electronic Supplementary Material

Structures and cytotoxicities of three new sesquiterpenes from cultures of *Armillaria* sp.

Xia YIN,[a,b] Tao FENG,[a] and Ji-Kai LIU[a,*]

[a]State Key Laboratory of Phytochemistry and Plant Resources in West China, Kunming Institute of Botany, Chinese Academy of Sciences, Kunming 650201, China

[b]University of Chinese Academy of Sciences, Beijing 100049, China

Received 29 September 2012; Accepted 25 October 2012

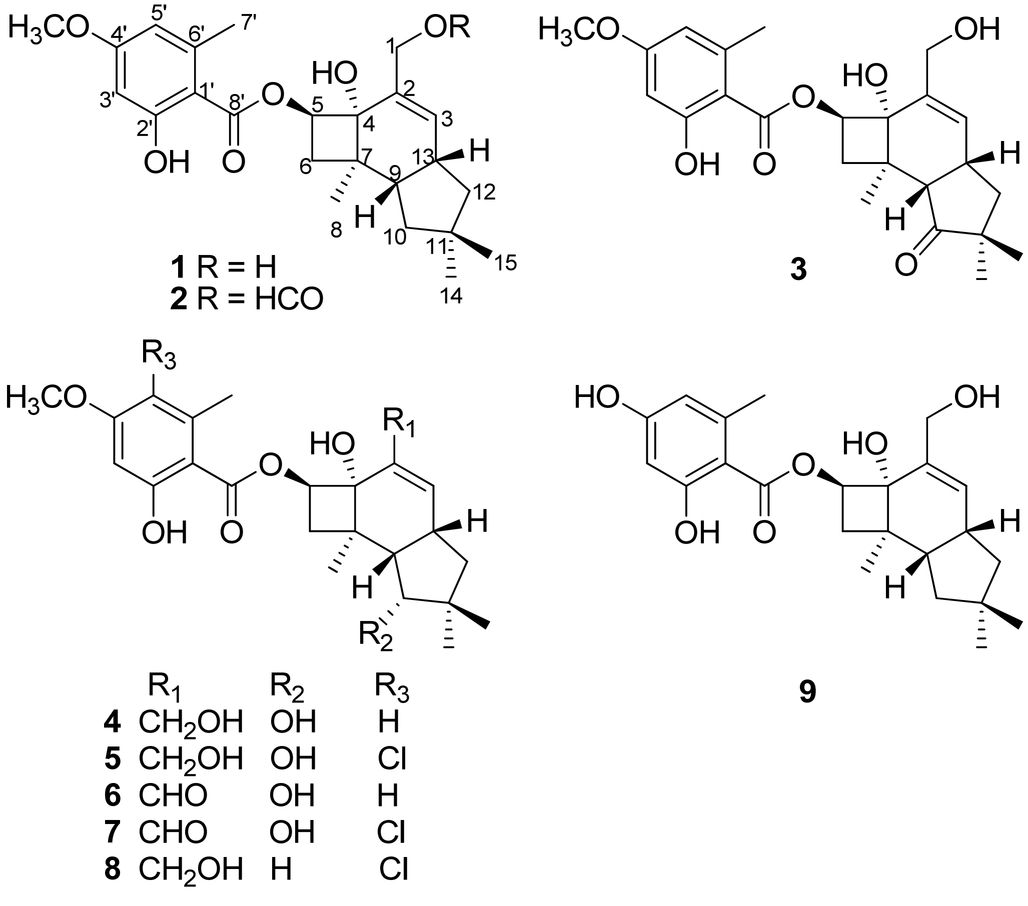

Structures of compounds **1**–**9**

*To whom correspondence should be addressed. E-mail: jkliu@mail.kib.ac.cn

Figure 1S-7S. NMR and MS spectra of **1**

Figure 8S-12S. NMR and MS spectra of **2**

Figure 13S-17S. NMR and MS spectra of **3**

Figure 1S. ^{1}H NMR of **1**

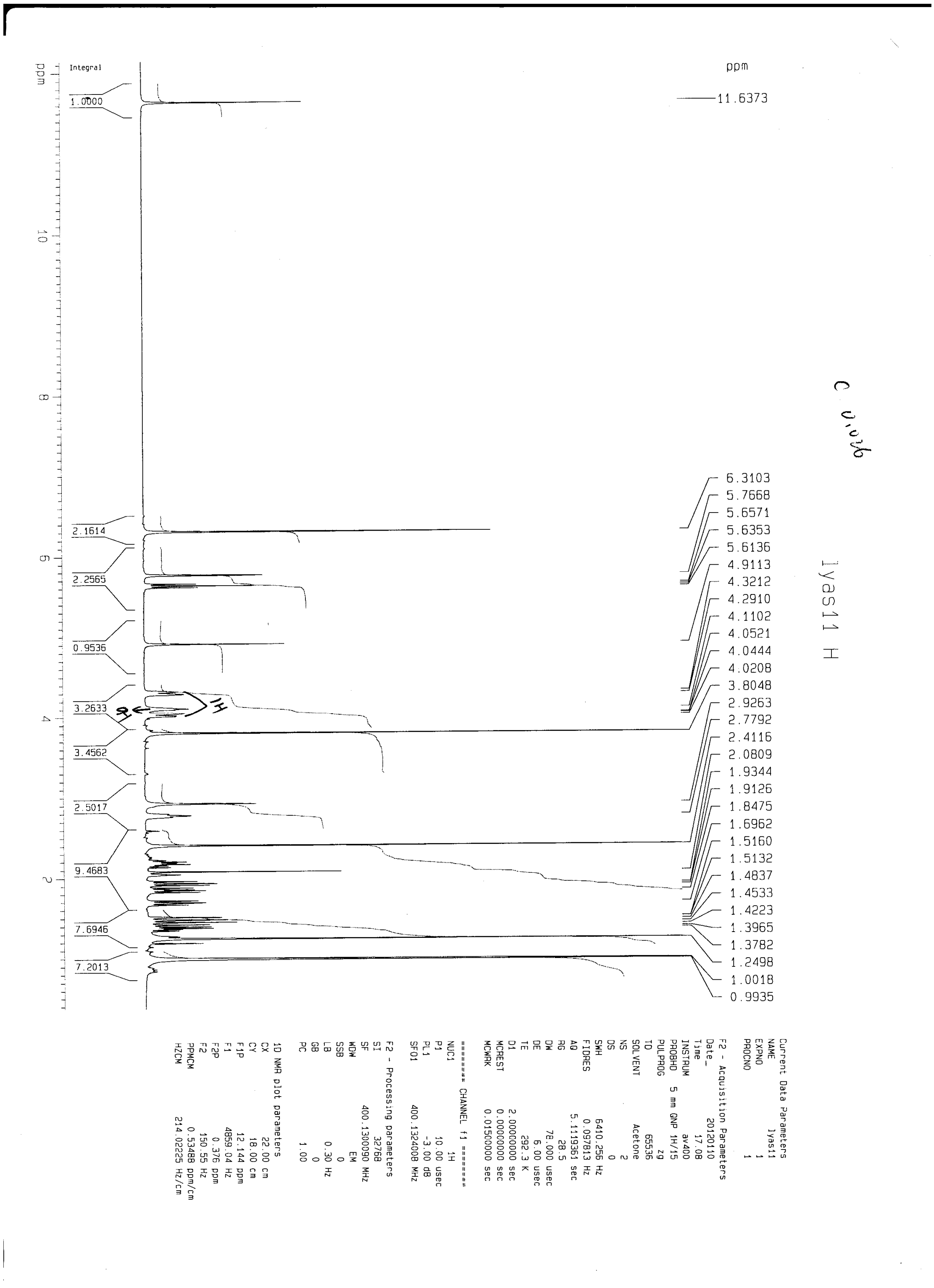

Figure 2S. ^{13}C NMR of **1**

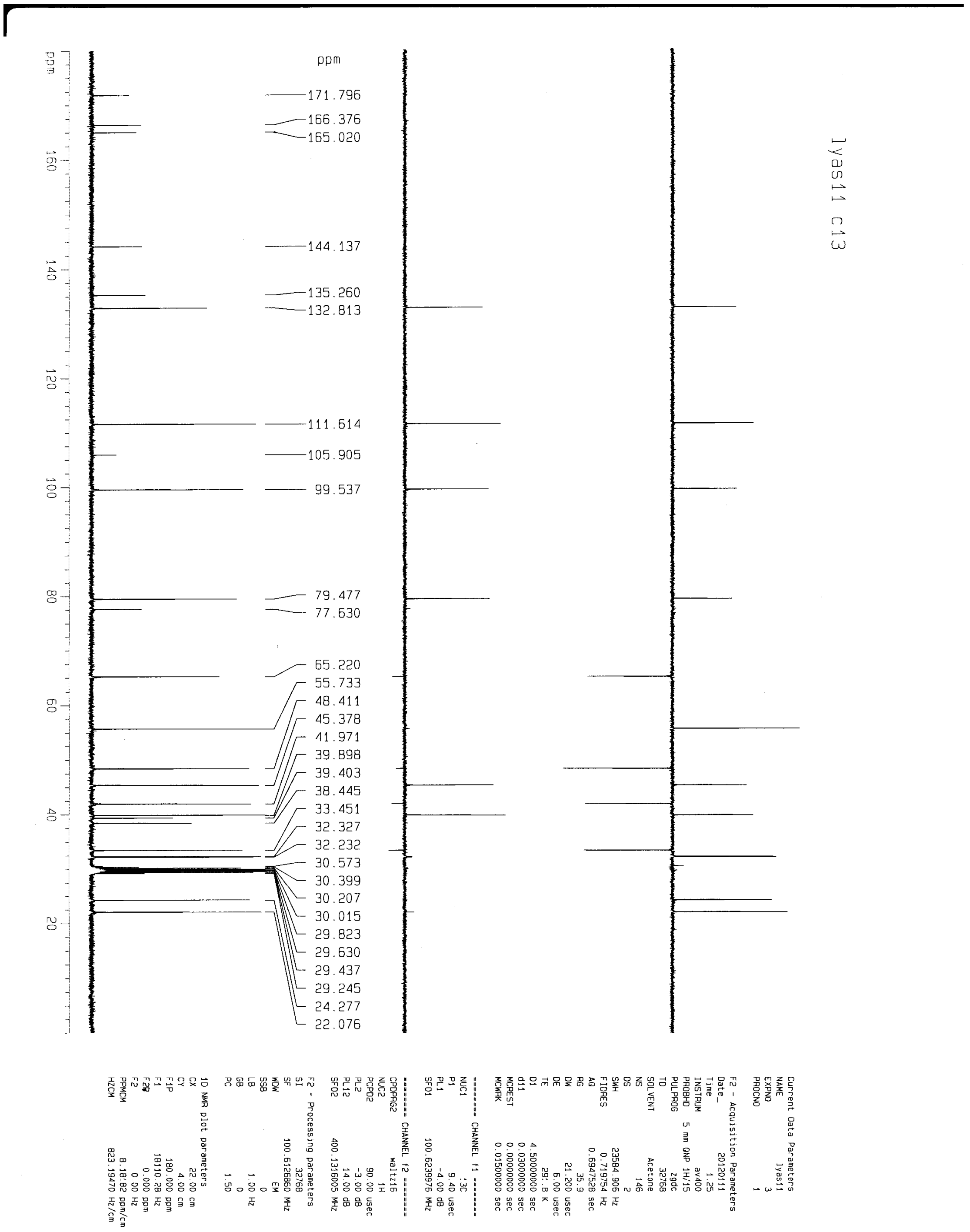

Figure 3S. HSQC of **1**

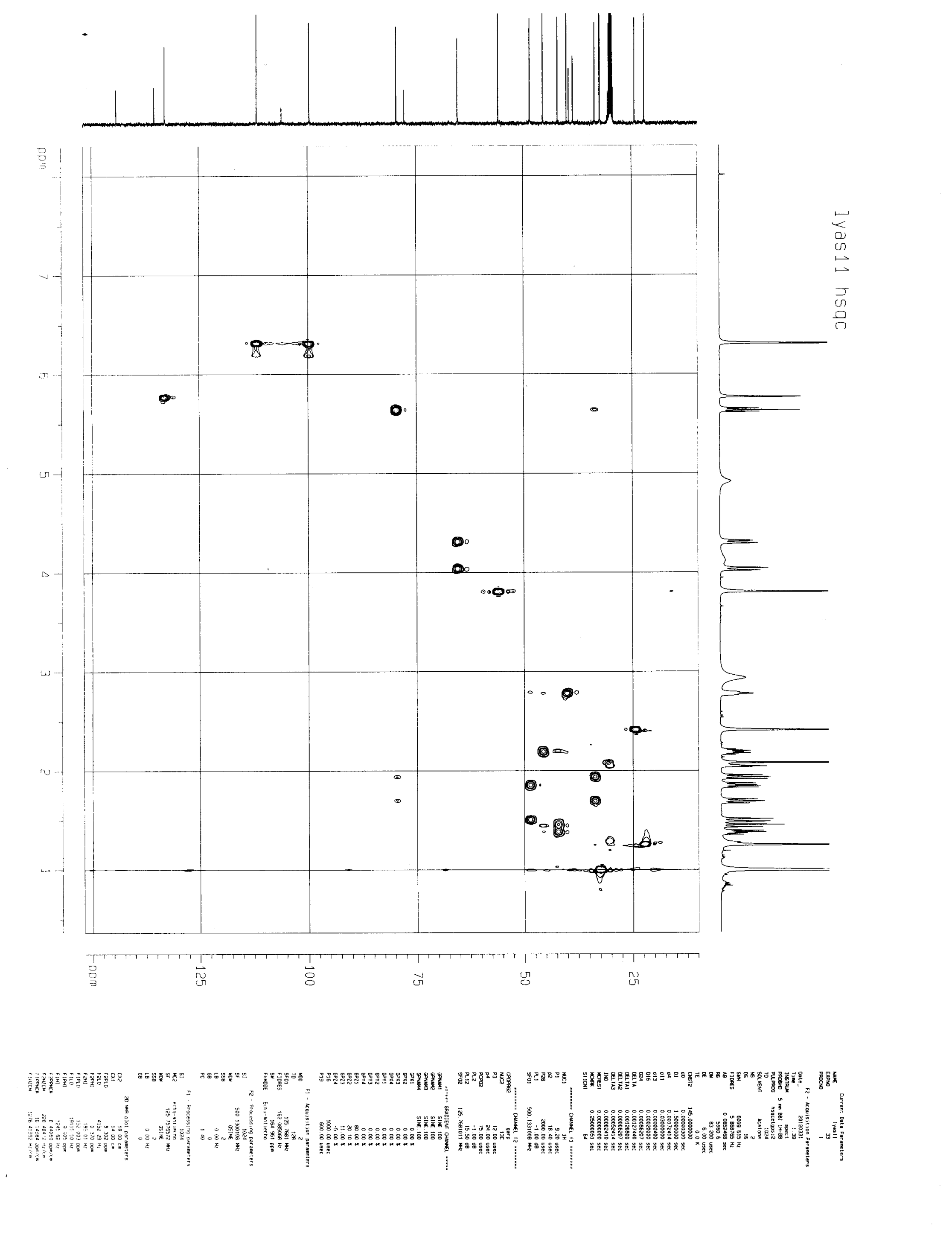

Figure 4S HMBC of **1**

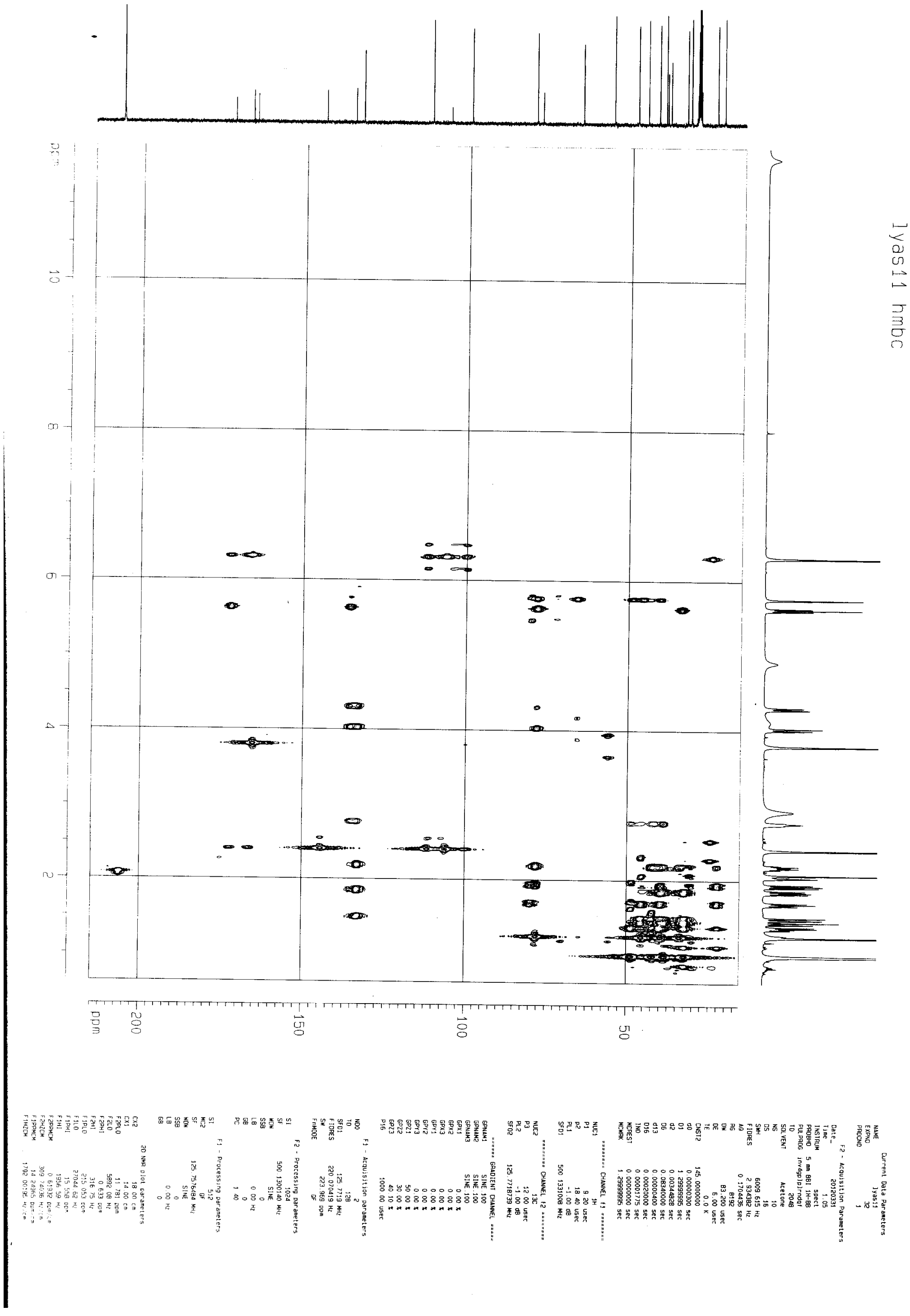

Figure 5S ^{1}H-^{1}H COSY of **1**

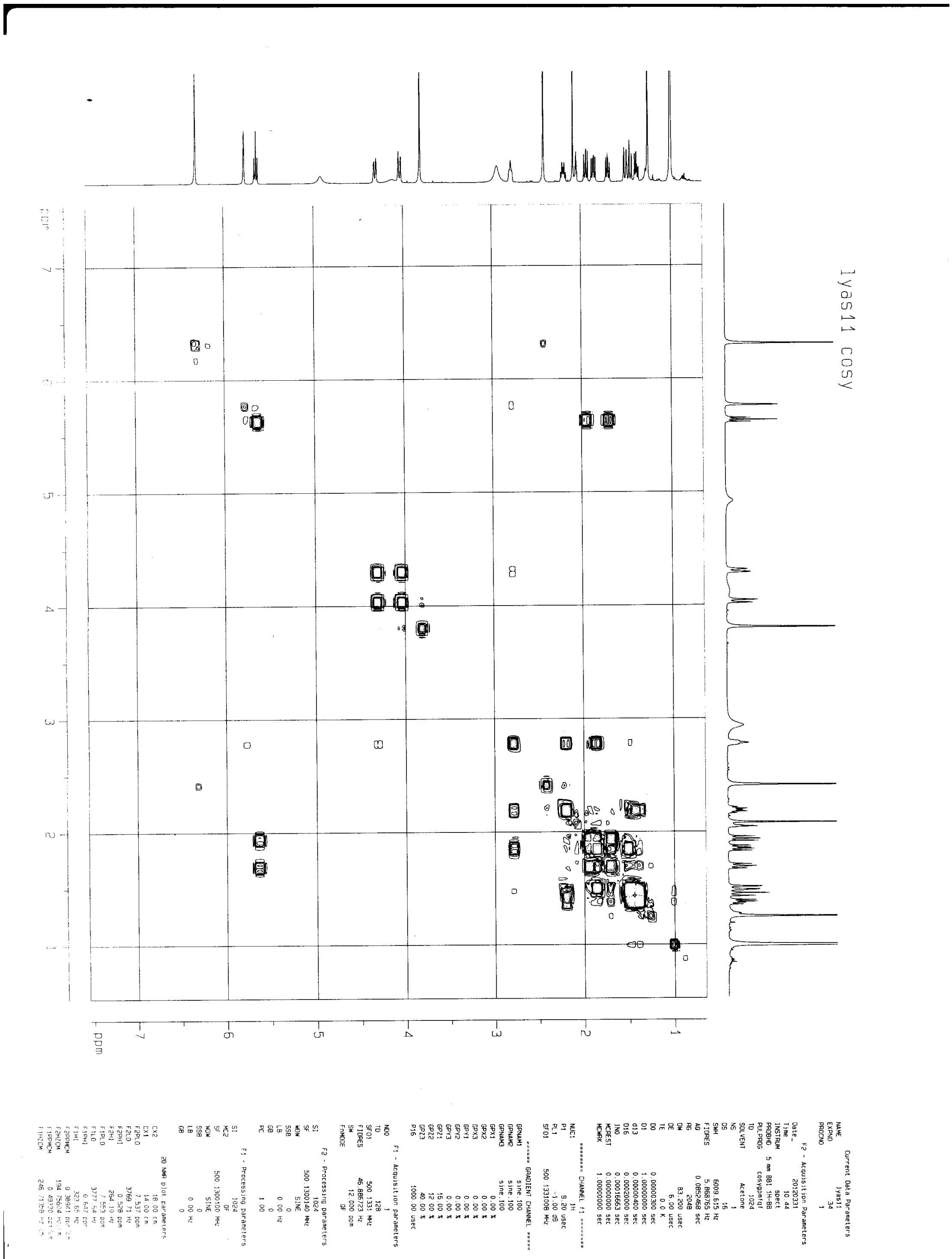

Figure 6S ROESY of **1**

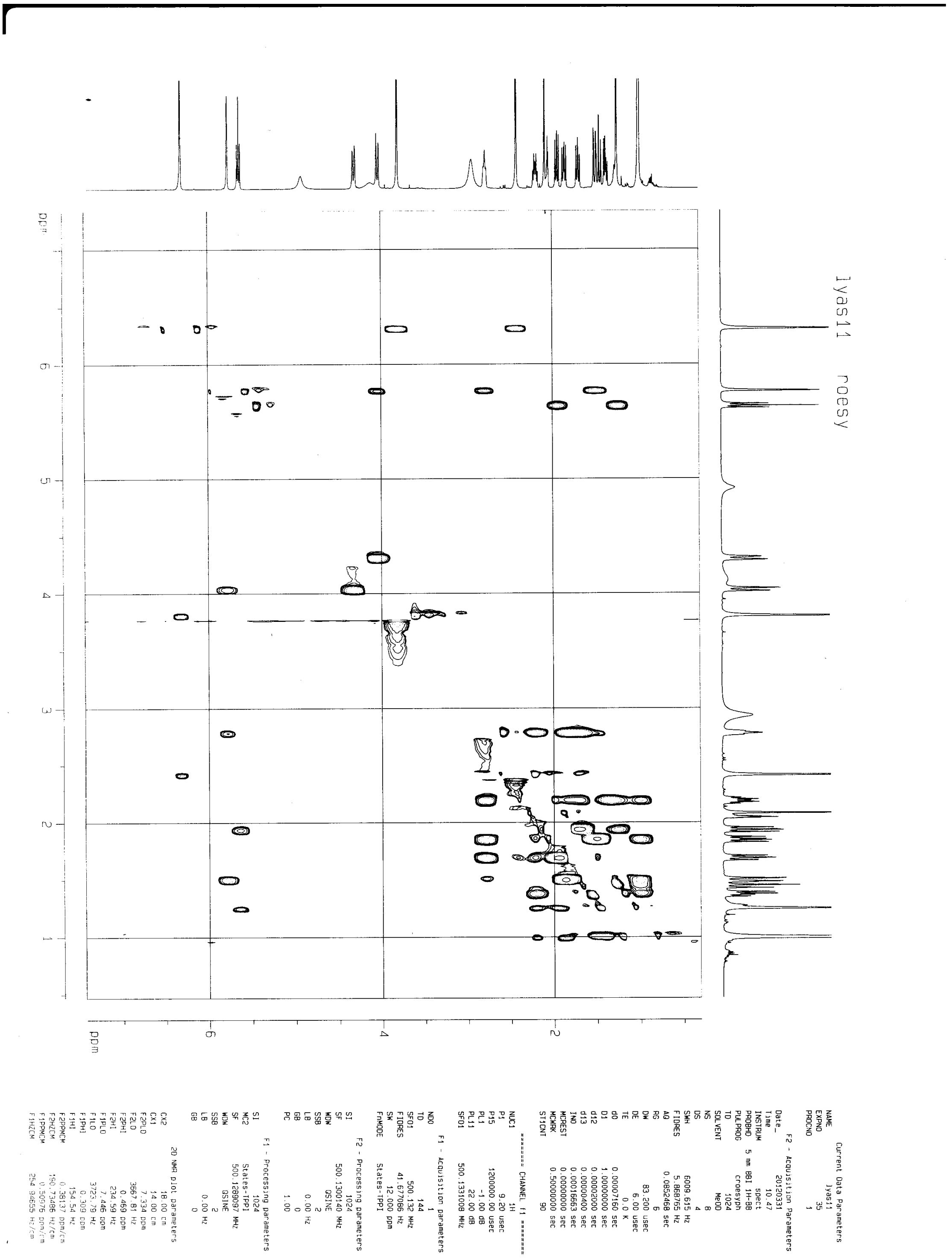

Figure 7S HRESIMS of **1**

Acq. Date: Thursday, March 01, 2012 *Acq. Time: 15:18* *Scan Mode: Zero Width*

Sample Name: 120301ESINA LYAS-11 *Sample Comment:*

Elemental composition calculator

Target m/z: +415.2118 amu
Tolerance: +10.0000 ppm
Result type: Elemental
Max num of results: 1000
Min DBE: -10.0000 Max DBE: +60.0000
Electron state: OddAndEven
Num of charges: 0
Add water: N/A
Add proton: N/A
File Name: 120301ESINA LYAS-11.wiff

	Elements	Min Number	Max Number
1	2H	0	0
2	Br	0	0
3	C	0	200
4	Cl	0	0
5	F	0	0
6	H	0	400
7	I	0	0
8	K	0	0
9	N	0	0
10	Na	0	0

Figure 8S。 ^{1}H NMR of **2**

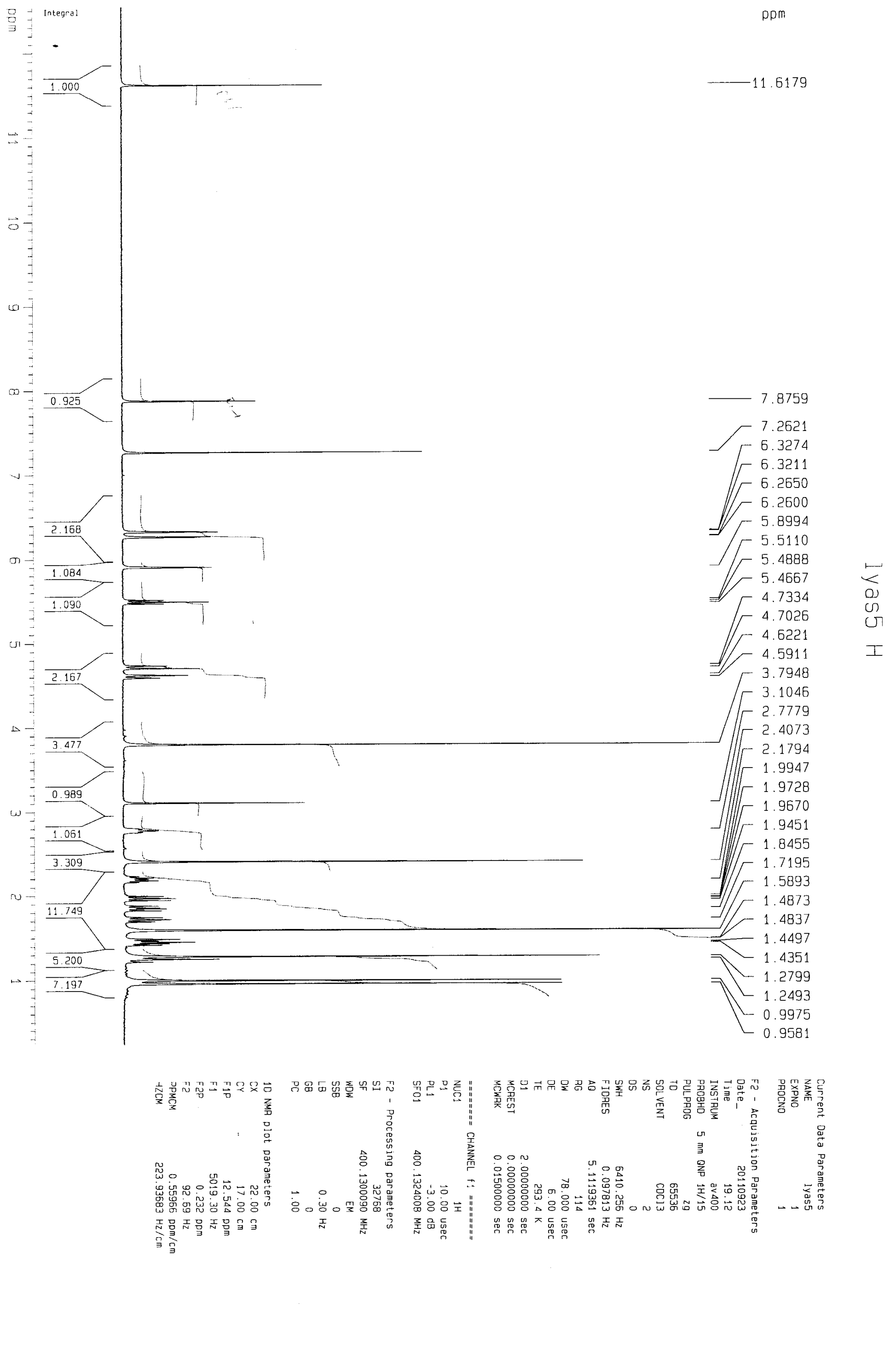

Figure 9S。^{13}C NMR of **2**

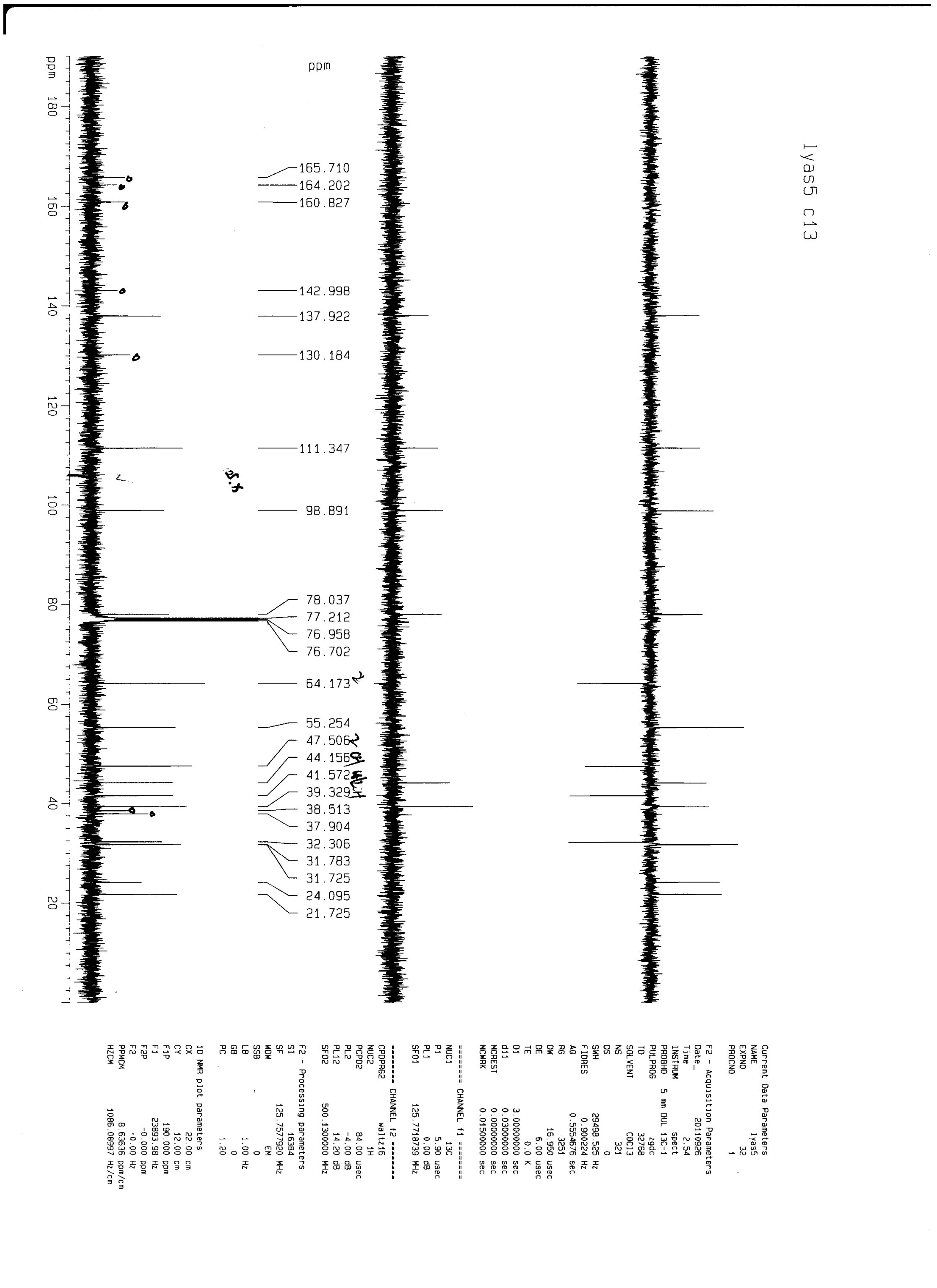

Figure 10S。HSQC of **2**

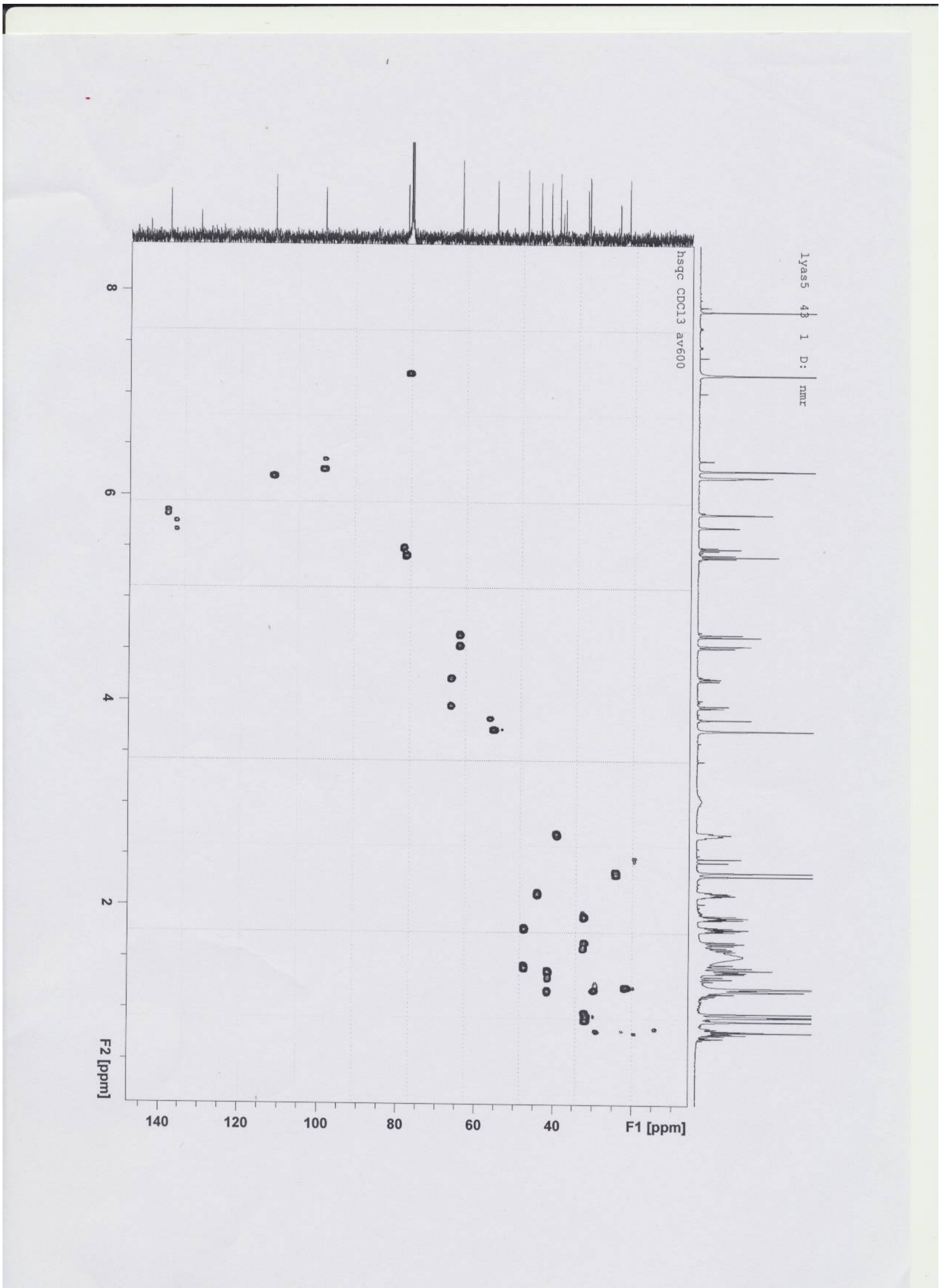

Figure 11S。HMBC of **2**

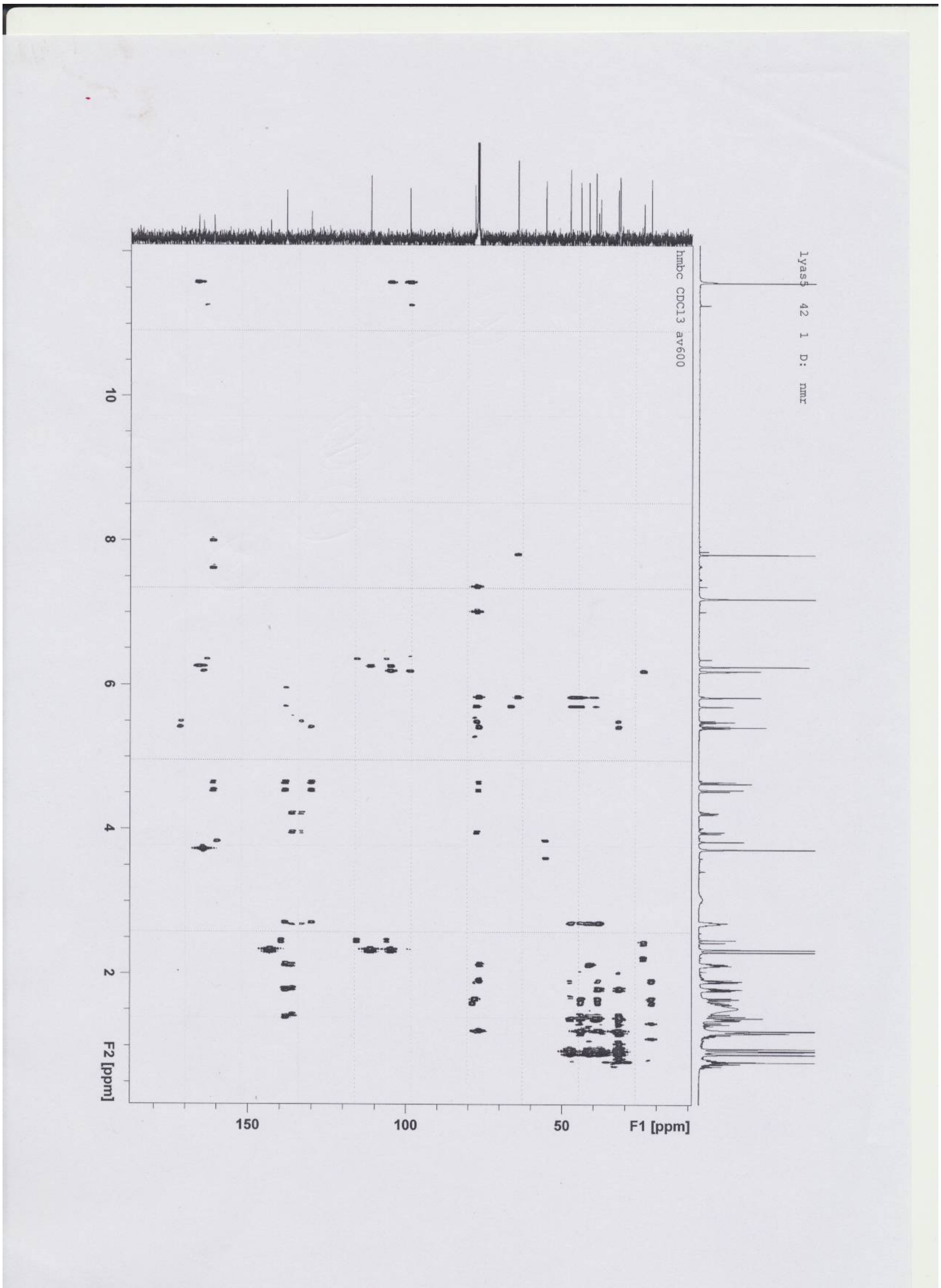

Figure 12S. HRESIMS of **2**

[illegible]day, March 01, 2012 | Acq. Time: 15:27 | Scan Mode: Zero Width

[illegible]e: 120301ESINA LYAS-5 | Sample Comment:

Elemental composition calculator

Target m/z: +443.2070 amu
Tolerance: +10.0000 ppm
Result type: Elemental
Max num of results: 1000
Min DBE: -10.0000 Max DBE: +60.0000
Electron state: OddAndEven
Num of charges: 0
Add water: N/A
Add proton: N/A
File Name: 120301ESINA LYAS-5.wiff

	Elements	Min Number	Max Number
1	2H	0	0
2	Br	0	0
3	C	0	200
4	Cl	0	0
5	F	0	0
6	H	0	400
7	I	0	0
8	K	0	0
9	N	0	0
10	Na	0	0

Figure 13S. ^{1}H NMR of **3**

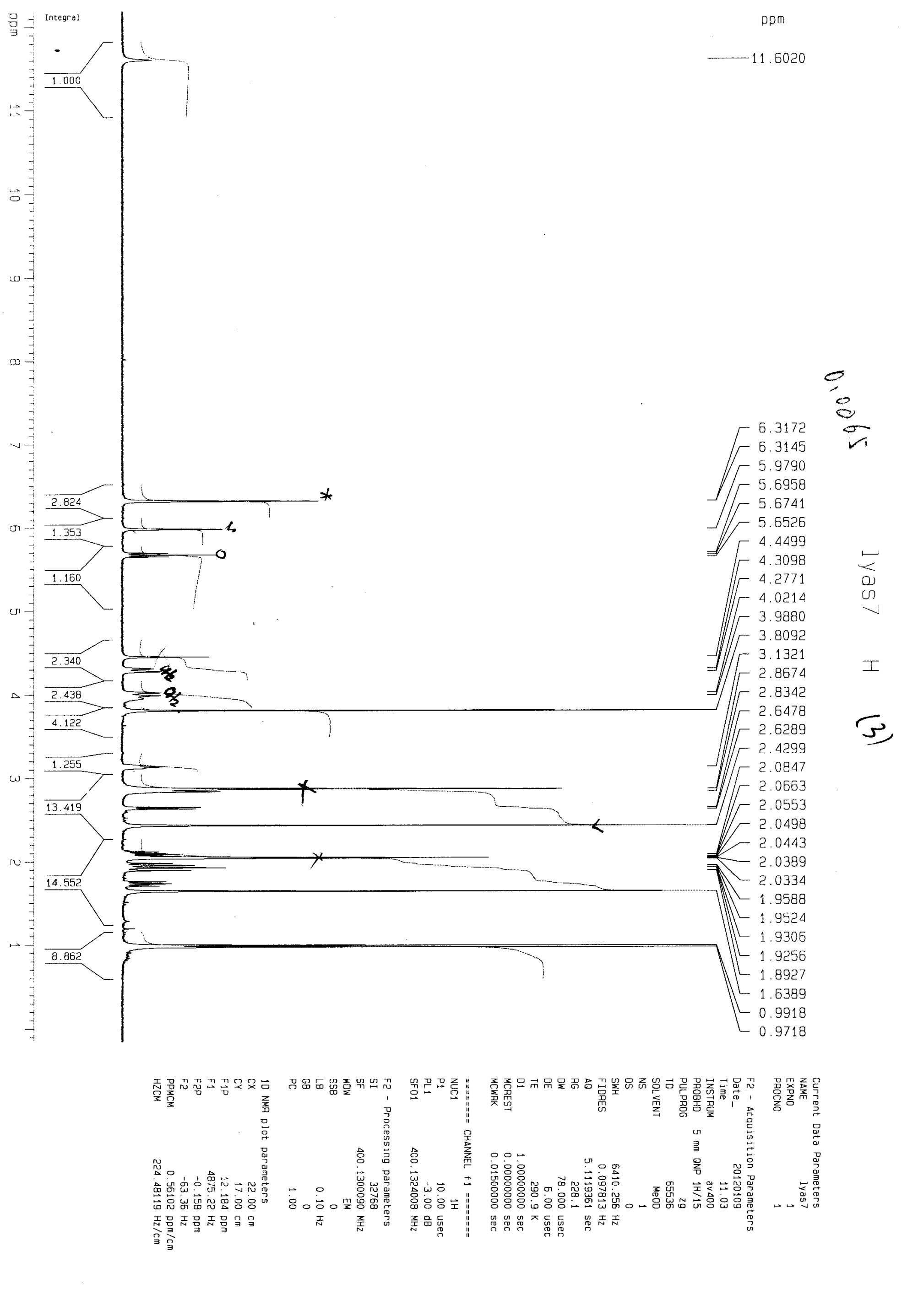

Figure 14S. ^{13}C NMR of **3**

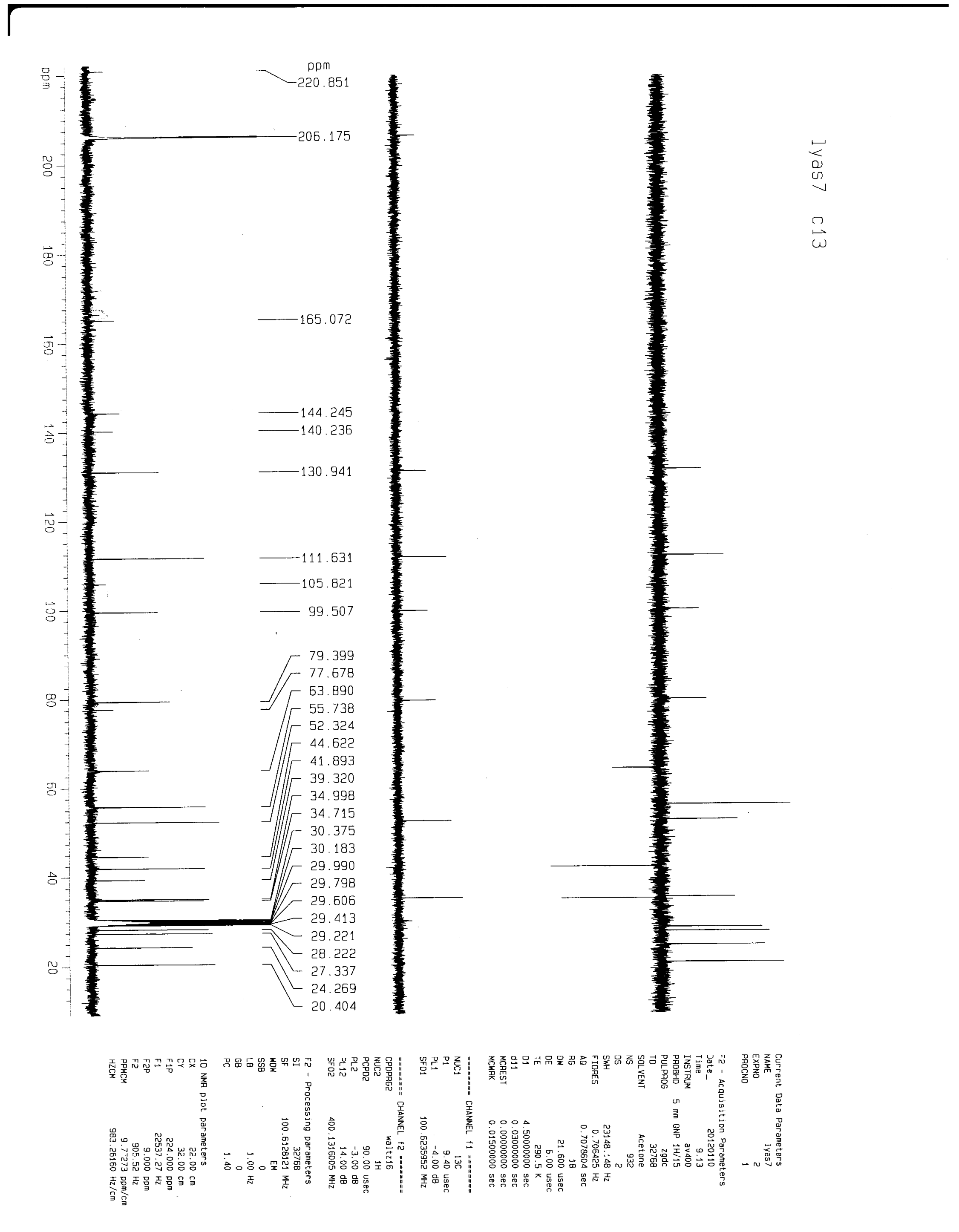

Figure 15S. HSQC of **3**

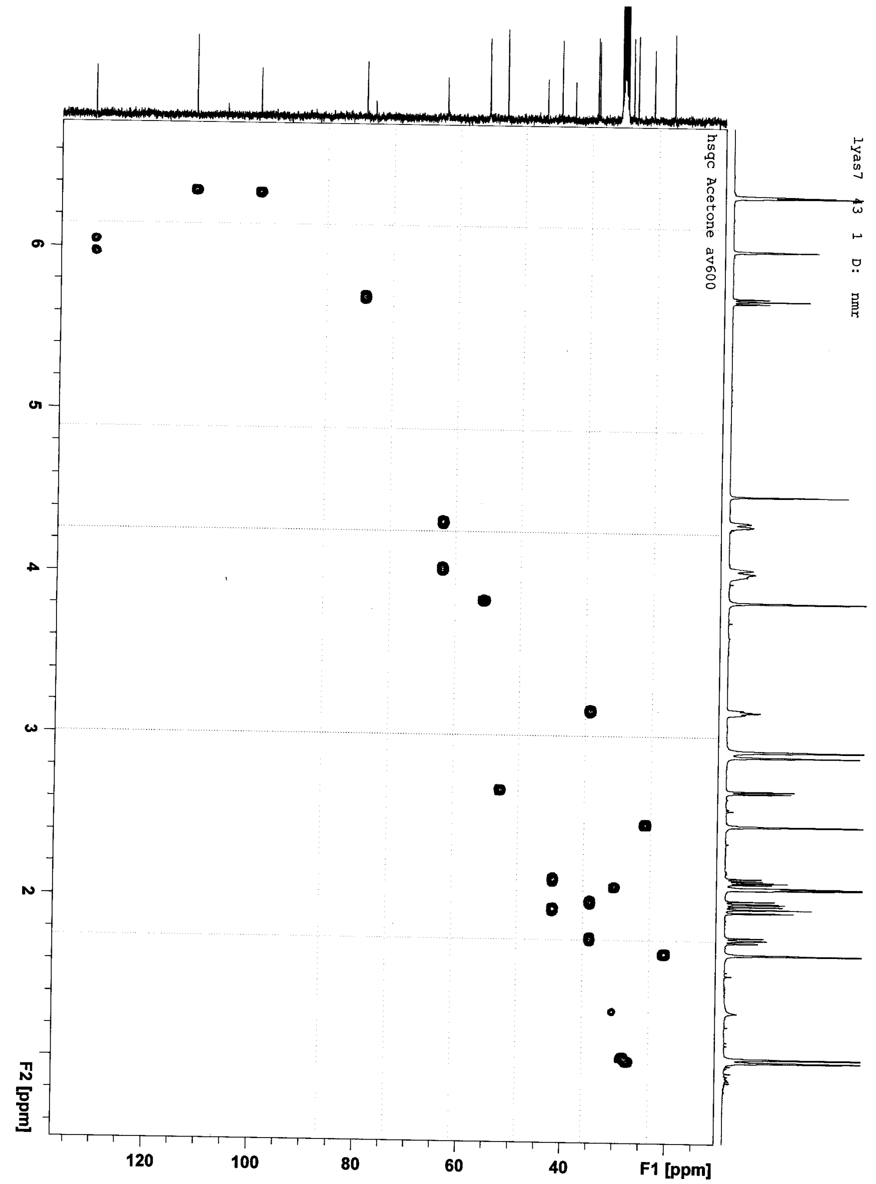

Figure 16S. HMBC of **3**

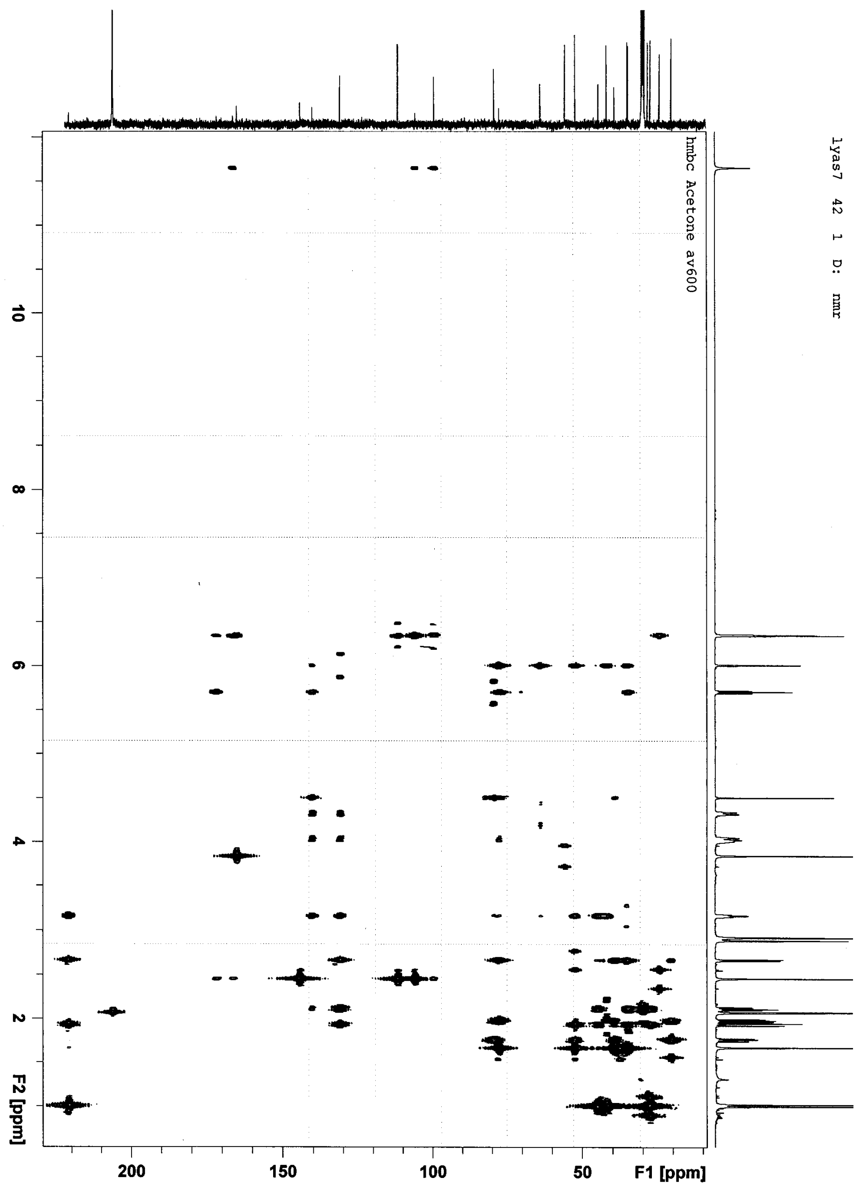

Figure 17S. HRESIMS of **3**

Acq. Date: Thursday, March 01, 2012 *Acq. Time: 15:24* *Scan Mode: Zero Width*

Sample Name: 120301ESINA LYAS-7 *Sample Comment:*

Elemental composition calculator

Target m/z: +429.1917 amu
Tolerance: +10.0000 ppm
Result type: Elemental
Max num of results: 1000
Min DBE: -10.0000 Max DBE: +60.0000
Electron state: OddAndEven
Num of charges: 0
Add water: N/A
Add proton: N/A
File Name: 120301ESINA LYAS-7.wiff

	Elements	Min Number	Max Number
1	2H	0	0
2	Br	0	0
3	C	0	200
4	Cl	0	0
5	F	0	0
6	H	0	400
7	I	0	0
8	K	0	0
9	N	0	0
10	Na	0	0
